# Gastroprotective Effect of Ethanolic Extract of *Curcuma xanthorrhiza* Leaf against Ethanol-Induced Gastric Mucosal Lesions in *Sprague-Dawley* Rats

**DOI:** 10.1155/2014/416409

**Published:** 2014-03-24

**Authors:** Nurhidayah Ab. Rahim, Pouya Hassandarvish, Shahram Golbabapour, Salmah Ismail, Saad Tayyab, Mahmood Ameen Abdulla

**Affiliations:** ^1^Department of Biomedical Science, Faculty of Medicine, University of Malaya, 50603 Kuala Lumpur, Malaysia; ^2^Medical Laboratory Technology, Faculty of Health Science, University Technology of MARA (Bertam Campus), 13200 Pulau Pinang, Malaysia; ^3^Institute of Biological Sciences, Faculty of Science, University of Malaya, 50603 Kuala Lumpur, Malaysia

## Abstract

Herbal medicines appeared promising in prevention of many diseases. This study was conducted to investigate the gastroprotective effect of *Curcuma xanthorrhiza* leaf in the rats induced gastric ulcer by ethanol. Normal and ulcer control received carboxymethycellulose (5 mL/kg) orally, positive control was administered with 20 mg/kg omeprazole (reference drug) and 2 groups were received 250 mg/kg and 500 mg/kg of the leaf extract, respectively. To induce of gastric ulcers formation, ethanol (5 mL/kg) was given orally to all groups except normal control. Gross ulcer areas, histology, and amount of prostaglandin E_2_, superoxide dismutase and malondialdehyde were assessed to determine the potentiality of extract in prevention against gastric ulcers. Oral administration of extract showed significant gastric protection effect as the ulcer areas was remarkably decreased. Histology observation showed less edema and leucocytes infiltration as compared with the ulcer control which exhibited severe gastric mucosa injury. Furthermore, the leaf extract elevated the mucus weight, level of prostaglandin E_2_ and superoxide dismutase. The extract also reduced malondialdehyde amount significantly. Results showed leaf extract of * Curcuma xanthorrhiza* can enhanced the gastric protection and sustained the integrity of gastric mucosa structure. Acute toxicity test did not showed any sign of toxicity (2 g/kg and 5 g/kg).

## 1. Introduction 

The mucosal layer of stomach secretes mucus that contains greasy substances to facilitate the food movement [1]. This layer provides a barrier to protect the stomach against the environmental irritations such as pepsin and hydrochloride acid which may cause diffusion of acid into gastric mucosa and may burst the mucosal blood vessels [2]. Secretion of gastric acid is induced by several factors such as osmotic pressure activity, volume, pH, and caloric control of the infusate [3]. Furthermore, gastric acid has the ability to interrupt several growth factors like fibroblast growth factor and epidermal growth factor which in turn may prevent the maintenance of mucosal integrity and restoration of superficial injury [2]. Gastric ulcers induced by alcohol may be characterized through releasing of various intermediates such as lipoxygenase, oxygen free radicals, and cytokines. Several studies [4–6] showed that administration of alcohol causes deterioration of gastric mucosa by increasing neutrophils infiltration, which then delays the healing process of ulcerated gastric tissues [7]. Moreover, alcohol produces reactive oxygen species (ROS) which can lead to cells death by cytotoxic effects and damage the tissues [8].

*Curcuma xanthorrhiza* belongs to the Zingiberaceae family. It is known as temulawak by the Malaysian community and as javanese turmeric in Indonesia. This plant is widely distributed in Southeast Asia countries such as Java, Peninsular Malaysia, Philippines, Thailand, and India [9]. This plant is traditionally used as a tonic in the treatment of gastrointestinal related diseases such as stomach cramps, less primitive, and slow digestion [10]. The main active compound that found in the roots is curcuminoids which is composed of curcumin, bisdemethoxycurcumin, and demethoxycurcumin [11]. Curcuminoids have antioxidant, anti-inflammatory, antibacterial, antifungal, antiparasitic, antimutagenic, anticancer, and detox properties. The presence of curcumin, bisdemethoxycurcumin, and demethoxycurcumin in the methanolic extract of* C. xanthorrhiza* is responsible to prevent joint inflammation such as rheumatoid arthritis [12]. These chemicals also exhibit antitumor properties through suppressing the harmful oxidants and thus inhibiting the cancer cell proliferation [13]. Rhizome of* C. xanthorrhiza* possesses xanthorrhizol which is able to inhibit the growth of* Staphylococcus aureus*,* Streptococcus mutans* type F, and* Bacillus cereus* [14]. Ethanolic extract of* C. xanthorrhiza *Roxb. is able to protect the liver from injuries through regulating the hepatoenzymes activities and strengthening the liver membrane [15].

The present study was carried out to investigate the gastroprotective mechanism of ethanolic extract of* C. xanthorrhiza* leaf against lesions in gastric mucosa layers induced by ethanol in experimental rats.

## 2. Materials and Methods

### 2.1. Omeprazole Drug

Omeprazole was used as a reference drug for antiulcer study and it was obtained from the Pharmacy of the University Malaya Medical Centre (UMMC). The drug was dissolved in 0.5% (w/v) carboxymethylcellulose and administered orally to the rats (20 mg/kg body weight, 5 mL/kg) according to the recommendations made by Abdulla et al. [16].

### 2.2. 0.5% Carboxymethylcellulose

Pure CMC (0.5 g) powder was mixed with distilled water (100 mL) and was stirred until clear solution formed.

### 2.3. Plant Specimen and Preparation of Ethanol Extract

Fresh* C. xanthorrhiza* leaf was obtained from the Herbarium of Rimba Ilmu, Institute of Biological Sciences, University of Malaya, Kuala Lumpur. The leaf was washed with distilled water and was dried in the oven at 50°C for five days. The dried leaf was powdered by an electrical blender (Panasonic, Japan). Hundred grams of the finest powder were soaked in 500 mL of 95% ethanol for 3 days. Then, the mixture was filtered with a fine muslin cloth followed by Whatman Number 1 filter paper and was distilled under reduced pressure in an Eyela rotary evaporator (Sigma-Aldrich, USA). The dried extract was kept in refrigerator (Panasonic, Japan) at −20°C. The dried extract was dissolved in 0.5% CMC at the doses of 2 g/kg and 5 g/kg for the acute toxicity study as well as 250 and 500 mg/kg for gastroprotection study.

### 2.4. Acute Toxicity Study and Experimental Animals

Healthy adult male and female* Sprague-Dawley* rats were used in this study. The rats (6–8 weeks of age and the weight is about 150–180 g) were purchased from the Animal House, Faculty of Medicine, University of Malaya (ethic reference number: PM/30/05/2012/NAR (R)). All females were nulliparous and nonpregnant. The rats were caged in groups of 6 rats, given tap water and standard pellet diet* ad libitum*. They were kept in the Animal Experimental Unit several days before the start of the treatment to allow for acclimatization. Thirty-six rats (18 males and 18 females) were randomly assigned equally into three groups: a vehicle group (0.5% CMC, 5 mL/kg, orally) [17] and two extract groups (2 g/kg and 5 g/kg of the extract, orally). Prior to the experiment, the rats were fasted overnight and food was withheld further 3 or 4 hours after the dosing. The rat was weighed and then administered accordingly by an oral gavage. The rats were monitored, especially during the first 24 hours for any sign of behavioral abnormalities and/or clinical abnormalities. Mortality was recorded, if any. The administered rats were followed up daily for 14 days. On the fifteenth day, the animals were sacrificed and subjected to necropsy for the gross examination, liver function, renal function tests, and histological evaluation. The study was approved by the Ethics Committee for Animal Experimentation, Faculty of Medicine, University of Malaya, Malaysia. All animals received human care according to the criteria outlined in the “Guide for the Care and Use of Laboratory Animals” prepared by the National Academy of Sciences and published by the National Institute of Health.

### 2.5. Experimental Animals for Gastric Ulcer

Healthy adult males of* Sprague-Dawley* rats weighing 200–250 g were purchased from the Experimental Animal House, Faculty of Medicine, University of Malaya, and passed the Ethical Clearance (ethic reference number: PM/30/05/2012/NAR (R)). The rats were maintained (temperature: 20–25°C) on standard pellet diets and tab water. However, they were kept deprived of food overnight before the experiments. The rats were housed individually with wide-mesh wire bottoms to prevent coprophagia during the experiment. The rats were divided randomly into 5 groups of 6 rats. The groups of were labeled as normal control, ulcer control, positive control (omeprazole), low dose (250 mg/kg), and high dose (500 mg/kg) of the leaf extract of* C. xanthorrhiza*, orally. All animals received human care according to the criteria outlined in the “Guide for the Care and Use of Laboratory Animals” prepared by the National Academy of Sciences and published by the National Institutes of Health [18].

### 2.6. Induction of Gastric Ulcer by Ethanol Administration

As previously done by Mahmood et al. [19], the rats were fasted 24 hours but drinking water was accessible until 2 hours before the experiment. Gastric ulcers were induced by orogastric intubation of absolute ethanol (5 mL/kg) according to the method described by Abdulla et al. [16]. Normal control rats were given 0.5% CMC (5 mL/kg). Ulcer control groups were orally administered with the vehicle solutions (0.5% CMC, 5 mL/kg). The reference (positive control) group received an oral dose of 20 mg/kg omeprazole dissolved in vehicle. Experimental groups were orally administered with the ethanolic extract of* C. xanthorrhiza *leaf dissolved in 0.5% CMC at dose of 250 and 500 mg/kg. One hour after, all of the groups except the normal control group were administered orally with absolute ethanol (5 mL/kg). The rats were euthanized 1 hour later with an overdose of xylazine and ketamine anesthetic drugs [20]. Their stomachs were separated and opened with an incision along the greater curvature. The gastric juice of each stomach was collected in a tube. The stomachs were rinsed with saline [21]. The luminal side of each stomach was examined grossly [18].

### 2.7. Gross Examination of Gastric Lesion

The gross examination of gastric mucosa showed that gastric ulcers mainly appeared as elongated bands of haemorrhagic lesions that were placed parallel to the long axis of the stomach. The length and width of each ulcer in mm were measured by a planimeter (10 × 10 mm^2^ = ulcer area) under dissecting microscope (1.8x) (Olympus, Japan). By covering the length and width of each ulcer band, the ulcerated area was measured by counting the number of small squares (2 mm × 2 mm). The sum of the areas of all lesions for each stomach was used in the calculation of the ulcer area (UA, mm^2^) which was given by the sum of small squares × 4 × 1.8. The inhibition percentage (*I*%) was calculated by the following formula according to the recommendations of Abdulla et al. [16]:

(1) $$\begin{array}{c} \left(I\%\right)=\left[\frac{{\text{UA}}_{\text{control}}-{\text{UA}}_{\text{treated}}}{{\text{UA}}_{\text{control}}}\right]\times 100\%. \end{array}$$

### 2.8. Measurement of Mucus Production

Gastric mucus production was measured in the rats. The gastric mucus of each rat was obtained by gently scraping the mucosa with a glass slide and the collected mucus was weighed by using a precision electronic balance (Sartorius, Germany) [20, 22].

### 2.9. Histological Evaluation of Gastric Lesion

Specimens of the gastric walls from each rat were cut into pieces and put into a plastic cassette. The stomach tissue was then fixed with 10% buffered formalin and processed in an automated tissue processing machine (Leica TP1020, Germany). The processed tissues were then infiltrated with paraffin (Paraplast Plus, Leica, Germany) using embedding machine (Leica EG1160, Germany) to produce tissue-paraffin embedding block. Sectioning of the stomach was accomplished by microtome (Leica LM2135, Germany) at a thickness of 5 *μ*m. The section was mounted on individual microscope slide. Then, tissues were stained with haematoxylin and eosin for histological evaluation.

### 2.10. Measurement of Prostaglandin E_2_ Activity

Using prostaglandin E_2_ (PGE_2_) monoclonal antibody, this assay was performed based on the reaction between PGE_2_ and a PGE_2_-acetylcholinesterase (AChE) conjugate, identified as PGE_2_ tracer. A distinct yellow color revealed the end product of this enzymatic reaction which is strongly absorbed at 412 nm. The amount of PGE_2_ was directly proportional to the intensity of color formed which was determined by a spectrophotometer. For the determination of PGE_2_ concentration, the entire gastric tissue samples were prepared by homogenizing it in 1.15% potassium chloride at a ratio of 1 : 5 (w/v) followed by centrifugation at 4°C. The resulting supernatant was centrifuged again at 4°C and the concentration of PGE_2_ was measured using a kit purchased from the Cayman Chemical Company, USA. The PGE_2_ concentration was determined by recording the absorbance at wavelengths between 405 and 420 nm using a plate reader (PowerWave X340, Bio-Tek Instruments, USA).

### 2.11. Measurement of Superoxide Dismutase Activity

For detection of superoxide radicals that are created by xanthine oxidase and hypoxanthine, this assay utilizes a tetrazolium salt as a detector. The superoxide dismutase (SOD) enzyme causes dismutation of the superoxide radicals. For the determination of SOD activity, the entire gastric tissue samples were prepared by homogenizing in 1.15% potassium chloride at a ratio of 1 : 5 (w/v) followed by centrifugation at 4°C. The resulting supernatant was centrifuged again at 4°C and the SOD activity was measured using a kit purchased from the Cayman Chemical Company, USA. The activity of the SOD enzyme was determined by recording the absorbance at wavelengths between 440 and 460 nm using a plate reader (PowerWave X340, Bio-Tek Instruments, USA).

### 2.12. Measurement of Membrane Lipid Peroxidation

This assay was based on the reaction between malondialdehyde (MDA) and thiobarbituric acid (TBA) producing an MDA-TBA_2_ compound which was strongly detected at a wavelength of 532 nm. The entire gastric tissue samples were prepared by homogenizing in 1.15% potassium chloride at a ratio of 1 : 5 (w/v) followed by centrifugation at 4°C. The resulting supernatant was centrifuged again at 4°C and MDA was measured using a kit purchased from the Northwest Life Science Specialties, LLC, Canada. The concentration of the MDA was determined by recording the absorbance at wavelengths between 400 and 700 mm using a plate reader (PowerWave X340, Bio-Tek Instruments, USA).

### 2.13. Statistical Analysis

All values were reported as mean ± S.E.M. The statistical significance of differences between groups was analyzed using one-way ANOVA, SPSS (version 17) software. A value of *P* < 0.05 was considered as significant.

## 3. Results 

### 3.1. Acute Toxicity Study

Two doses of ethanolic leaf extract of* C. xanthorrhiza* (2 g/kg and 5 g/kg) were administered to the rats accordingly to identify the dose limit which is safe to use. During the period of 14 days, no significant toxic signs or death was recorded. Each rat showed no clinical toxic signs such as jaundice, depression, anorexia, dermatitis, lethargy, hyperactivity, and hypoactivity. The biochemical parameters for liver functions and renal functions appeared in their respective normal range. The result of biochemical test of* C. xanthorrhiza* leaf extract is presented in Tables 1 and 2.

**Figure 1:**
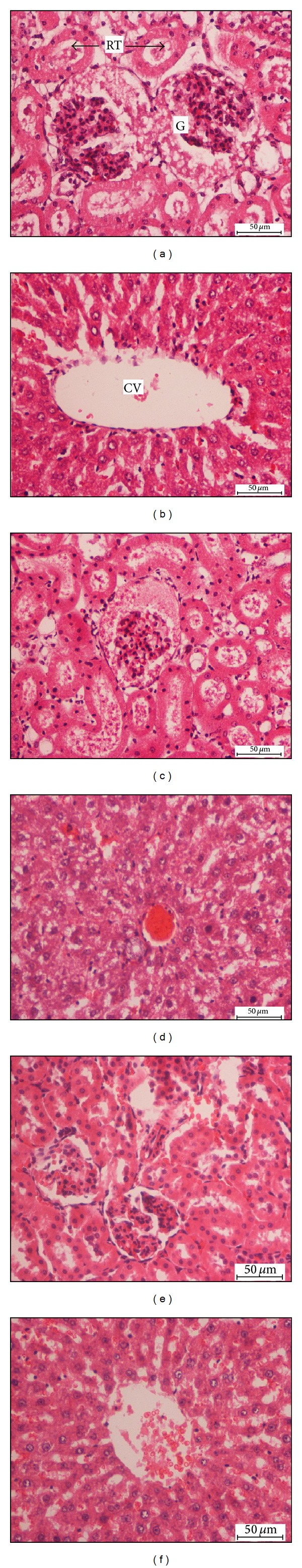
Histological sections of kidney ((a), (c), (e)) and liver ((b), (d), (f)) tissues obtained from experimental rats in acute toxicity study. ((a) and (b)) Rats were pretreated with the vehicle (0.5% CMC, 5 mL/kg); ((c) and (d)) rats were pretreated with the low dose of* C. xanthorrhiza *leaf extract (2 g/kg); ((e) and (f)) rats were pretreated with the high dose of* C. xanthorrhiza *leaf extract (5 g/kg). No pathological lesions and significant differences in structure of kidney and liver between leaf extract and control group were observed. (Haematoxylin and Eosin stain, 40x magnifications). Microphotograph shows central vein (CV), renal tubules (RT), and glomeruli (G).

The histological assessment for kidney and liver revealed no abnormalities when compared to the respective normal tissues. There were no structural alterations and the tissues of treated organs showed similar structure to the control group of rats (Figure 1).

### 3.2. Gross Examination of Gastric Lesions

**Figure 2:**
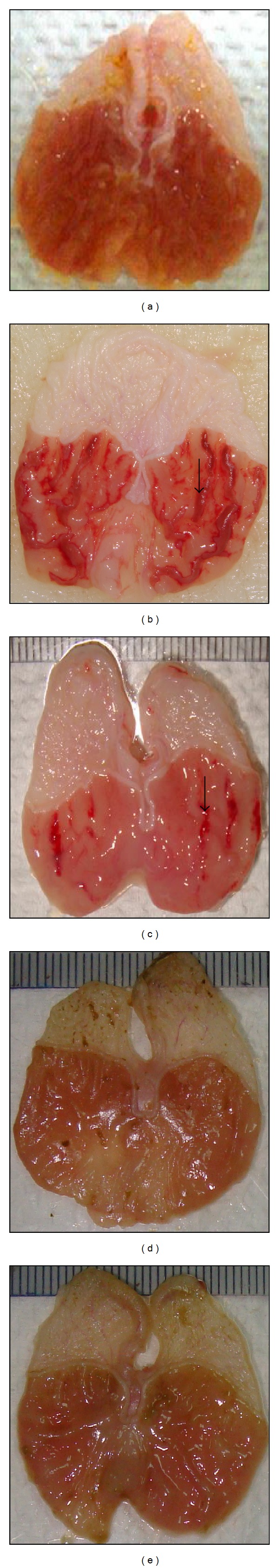
Gross examination of the gastric mucosa in rats. (a) Untreated rats (normal control). Intact gastric mucosa tissues are seen; (b) rats pretreated with 0.5% CMC (5 mL/kg, ulcer control). Severe lesions are seen with extensive visible haemorrhagic necrosis of gastric mucosa; (c) rats pretreated with omeprazole (20 mg/kg, positive control). Mild lesions of gastric mucosa are observed compared to the lesions in ulcer control group; (d) rats pretreated with low dose of* C. xanthorrhiza *leaf extract (250 mg/kg); (e) rats pretreated with high dose of* C. xanthorrhiza *leaf extract (500 mg/kg). No lesions are formed which indicates full protection of plant extract against gastric ulcers. The black arrow indicates the presence of gastric lesions.

An ulcer control group produced severe damage and extensive visible haemorrhagic necrosis of gastric mucosa due to the induction of ethanol (Figure 2(b)). The positive control group showed less injury with significantly reduced areas of gastric ulcer formation compared to ulcer control group. No haemorrhagic bands of ulcers or injuries were observed in the gastric mucosa when the rats were pretreated with the leaf extract of* C. xanthorrhiza* at low (250 mg/kg) and high (500 mg/kg) dose (Figures 2(d) and 2(e)). This plant extract manifested a significant protection (100% ulcer inhibition) against ethanol-associated gastric ulcers in rats.

### 3.3. Histological Evaluation of Gastric Lesion

**Figure 3:**
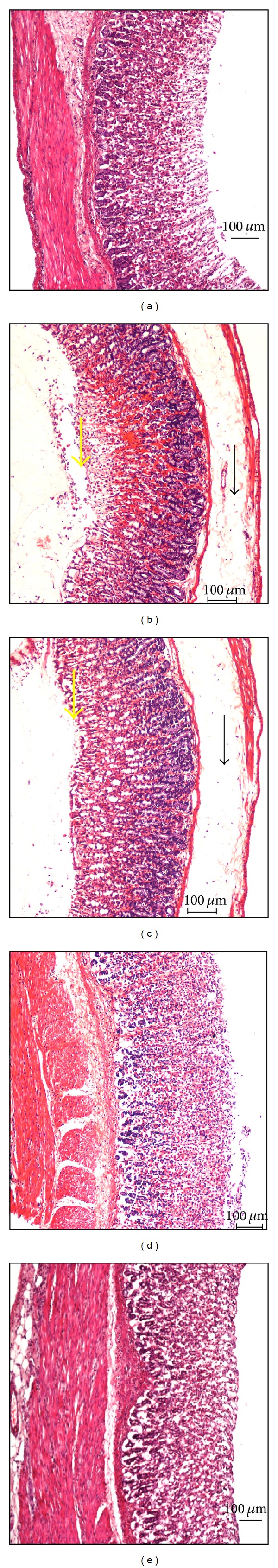
Histological evaluation of gastric mucosa in the ethanol-induced ulcerated rats. (a) Rats pretreated with 0.5% CMC only (normal control). Intact gastric mucosa layer are seen; (b) rats pretreated with 0.5% CMC (5 mL/kg, ulcer control). There is severe destruction to surface epithelium and necrotic lesions are present; (c) rats pretreated with omeprazole (20 mg/kg, positive control). Less disruption to gastric mucosa layer with mild leucocyte infiltration and edema; (d) rats pretreated with low dose of* C. xanthorrhiza* leaf extract (250 mg/kg). No disturbance to gastric mucosa layer; (e) rats pretreated with high dose of* C. xanthorrhiza* leaf extract (500 mg/kg). No disturbance to gastric mucosa layer. The black arrow indicates leucocytes infiltration and edema in submucosal layer; yellow arrow indicates disruption to the surface epithelium and deep mucosa (Haematoxylin and Eosin stain, 40x).

The histological result showed that the rats pretreated with omeprazole had markedly better protection of gastric mucosa by mild leucocyte infiltration and edema in submucosal layer as well as less disruption to the surface epithelium and deep mucosa (Figure 3(c)). In comparison, rats in the ulcer control group represented severe destruction of the surface epithelium and necrotic lesions penetrating deeply into mucosa as well as severe edema of submucosa layer. In addition, leucocyte infiltration was present too (Figure 3(b)). The pretreatment of rats with* C. xanthorrhiza* leaf extract did not show any significant leucocyte infiltration, edema, and disruption of deep mucosa (Figures 3(d) and 3(e)). Gastric mucosa tissue pretreated by this leaf extract showed intact appearance of histological structure as a normal control group.

### 3.4. Effect of* C. xanthorrhiza* Leaf Extract on the Production of Mucus Content in Gastric Mucosa

The presence of mucus in gastric mucosa play an important role as a diffusion barrier and lubrication to protect the stomach from harmful agents, suppress the growth of microorganisms and facilitate the movement of food through the gastrointestinal tract [23]. The mucus content of gastric mucosa in rats treated with ethanol (ulcer control group) was significantly reduced when compared to positive control or the extract groups. The pre-treatment of rats with low and high doses of* C. xanthorrhiza *leaf extracts significantly elevated the mucus content in the ethanol-induced ulcerated rats (Table 3). The increases of mucus production revealed that the leaf extract had potential to induce the mucus secretion and thereby protect the stomach layer from any injury caused by noxious and other agents.

### 3.5. Effect of* C. xanthorrhiza* Leaf Extract on the Production of PGE_2_ in Gastric Mucosa

PGE_2_ has been recognized as an agent that can induce the synthesis and secretion of mucus to prevent any damage in the gastric mucosa surface [24]. The result showed that the synthesis of PGE_2_ was significantly higher in the groups of rats pretreated with* C. xanthorrhiza* leaf extracts (Table 3).

### 3.6. Effect of* C. xanthorrhiza* Leaf Extract on Total SOD Activity in Gastric Mucosa

SOD is an enzyme that protects the cells from the irritable action of ROS such as superoxide [25]. Superoxide is capable of causing microvascular injury in the intestine and stomach as well as inducing gastric mucosal injury [26]. In the ulcer control group, the amount of SOD activity was significantly reduced compared to positive control group. The SOD activity was elevated in the group of rats pretreated with low and high dose of the leaf extract (Table 3). This result showed that the studied leaf extract could enhance the production of SOD enzymes, hence preventing gastric mucosa injury caused by ROS which was produced by ethanol.

### 3.7. Effect of* C. xanthorrhiza* Leaf Extract on the Amount of MDA in Gastric Mucosa

MDA is an active aldehyde and it is yielded from the reaction of ROS in the body [27]. The level of MDA was used to identify the presence of oxidative stress in the exposed group. The level of MDA was significantly higher in the ulcer control group but it was significantly reduced in the positive control group. MDA level was much lower in the rats pretreated with high dose (500 mg/kg) of* C. xanthorrhiza* leaf extracts compared to low dose (250 mg/kg) of this leaf extract (Table 3). The higher concentration of leaf extracts was able to reduce more oxidative stress process in the rats.

## 4. Discussion

For decades, researchers have been investigating herbs to find their effective active ingredients in the treatment of different diseases [28]. The acute toxicity study of* C. xanthorrhiza *leaf extracts showed nontoxic nature of the extract for the dose of <5 g/kg during 14 days. The animals behaved normally similar to the normal with no irritation, restlessness, respiratory distress, abnormal locomotion, and catalepsy. The level of alanine aminotransferase (ALT) and aspartate aminotransferase (AST) was used to assess the liver function [29]. Liver damaged or dysfunction such as impaired hepatocellular release more ALT and AST into the blood stream [30]. Another conventional indicator of liver damage is secretion of plasma proteins such as total protein, albumin, and globulin. The lower the level of these parameters, the more the incidence of chronic liver damage [31]. The biochemical indicators for the renal damages are serum urea, creatinine, and electrolytes such as sodium, potassium, bicarbonate, and chloride ions. In a normal kidney, the urea and creatinine are excreted out of the body whereas reabsorption of electrolytes takes place in the renal tubules. But, in the renal cellular failure and nephrotoxicity, the excretion of urea and creatinine is impaired accompanied by less reabsorption of electrolytes in the renal tubules. The nephrotoxicity is suggested to be associated with the low level of electrolytes and increased level of urea and creatinine [32]. In the present study, no significant changes in liver function and kidney parameters were identified in the rats pretreated with* C. xanthorrhiza *leaf extract. No structural abnormalities were found in liver and kidney tissues. Moreover, the LD_50_ value of this leaf extract was exceeded 5 g/kg.

For several years, the ethanol-induced gastric ulceration has been used as suitable method to study gastric ulcer [33–35]. Gastroprotective studies showed that ethanol could injure the epithelium of stomach and disrupt the vascular endothelium [4–6]. Ethanol may increase the permeability of the vessels and develop edema in submucosal layer of the stomach as well as epithelial lifting [36]. Ethanol also caused dissolution of mucus constituents and reduced the mucus contents. These changes would elevate the flow of sodium and potassium ions into the lumen and pepsin secretion. Furthermore, ethanol was able to trigger direct toxic effect on the body and indirectly alter the mucosal flow in gastric mucosa by increasingly transcapillary fluid filtration, and finally the epithelial lining was ruptured [37]. The result showed that the rats pretreated with* C. xanthorrhiza *leaf extracts significantly reduced gastric ulcer and increased cytoprotective effects, whereas the ulcer control group showed severe gastric haemorrhage as ethanol treatment would cause zonal necrotizing mucosal lesions and the formation of long ulcers as well as petechial lesion in gastric mucosa [38]. In the histological evaluation, as previously showed in various studies [4–6], acute ulcers were characterized by the presence of necrosis, granulation tissue, and hemorrhagic, while the chronic ulcers also have a granulation tissue at the base which is rich in blood vessels and macrophages as well as disruption of muscularis propria due to underlying fibrosis. This fibrosis is usually consisted of amorphous debris [39, 40]. In the present study, extract-pretreated rats showed lack of submucosal area of gastric mucosa layer with no edema and no leukocyte infiltration. Frisoli et al (2000) reported that the thickened submucosal layer seems associated with submucosal edema or haemorrhagic process and the presence of this process may lead to the development of acute gastrointestinal disease [41]. This result showed that the leaf extract was able to preserve the mucosal layer of gastric from harmful agents like ethanol. The thickening of submucosal layer was observed in the ethanol control group as ethanol caused edema and haemorrhagic lesions in mucosal layer. The active immune cells found in granulation tissue are macrophages and neutrophils. Neutrophils are a reservoir of inflammatory mediators and they could generate significant ROS such as hydrogen peroxide, superoxide, and myeloperoxidase. The excessive amount of ROS would be harmful to our body. These ROS are highly toxic to cells and lead to the tissue damage as well as tissue death. In addition, the ROS were able to delay the healing process of gastric ulcers. The administration of absolute ethanol into the body can elevate the ROS production [42] and thereby cause severe destruction in the gastric mucosa layer. Finally, the neutrophils infiltration into gastric mucosa was increased [8, 43].

Prostaglandin is a major component of the protective factors that maintain gastrointestinal mucosal integrity and microcirculation [44]. Prostaglandins could reduce the damage caused by necrotizing agents (such as alcohol, aspirin, bile salts) in the gastric mucosal barriers [45]. Prostaglandin could also elevate the mucus gel layer and maintain the pH gradient as well as increasing mucus viscosity [46]. In addition, the prostaglandin would inhibit the movement of acid and pepsin into the mucus layer. To prevent the diffusion of hydrogen ions into the gastric mucosa, bicarbonate is required to regulate mucus pH to an optimized acidity of the stomach that served as barrier for acid component [47]. A study in humans showed that acute alcohol ingestion at concentration of 12.5% has reduced the PGE_2_ production in gastric juice [48]. In the present study, the level of PGE_2_ was elevated in gastric mucosa due to the administration of* C. xanthorrhiza* leaf extract. In ulcer control group, the level of PGE_2_ dropped as ethanol administration at higher concentration may reduce the synthesis of PGE_2_ in gastric mucosa.

SOD is an enzyme found in the body and it could catalyze the dismutation of two superoxide radicals into two byproducts, hydrogen peroxide (H_2_O_2_) and oxygen (O_2_). SOD is essential to the body in order to discharge the harmful ROS from the cellular environment [49–51]. These radicals were able to attack major cellular macromolecules (lipids, proteins, DNA) and caused direct destruction of cell membranes, abnormality in the activation or inactivation of enzymes, and extensive mutations on the gene structure [52]. Superoxide was originated from the reduction process of oxygen, it can be catalyzed by enzyme for conversion to H_2_O_2_, or it can react with nitric oxide to produce peroxynitrite which is toxic to the body. The resulting product is capable of forming highly toxic hydroxyl radicals either by bonding between H_2_O_2_ and metal ions or breakage of peroxynitrite [53]. The group of rats that treated with* C. xanthorrhiza *showed significantly increased for SOD activity compared with ulcer control group.

Lipid peroxidation contributed to the pathogenesis of gastric ulcer which involved the destruction of membrane phospholipid and cell injury. Lipid peroxidation occurs when activated ROS attack unsaturated fatty acids of cell membrane phospholipids [54]. MDA is an organic compound that resulted from the degradation of polyunsaturated lipid. MDA is a highly reactive substance and toxic to the living cells. MDA used as a biomarker to determine the level of oxidative stress in an organism [27]. MDA synthesis is parallel with the process of lipid peroxidation [55]. The present study showed increased levels of MDA and decreased level of mucus contents of gastric mucosa in the ulcer control group. This process led to aggressive action on the developments of gastric ulcers. The decreased level of MDA in the rats pretreated with* C. xanthorrhiza* leaf extract and positive control group exhibited cytoprotective action in the ethanol-induced gastric ulcer rats.* C. xanthorrhiza* leaf extract had a greater reduction of MDA level showing a high defensive effect against gastrointestinal damage.

## 5. Conclusion 

We conclude that leaf extract of* C. xanthorrhiza* was safe to use at a dose up to 5 g/kg with no toxicity signs. This extract exhibited protective effects on the gastric mucosa by increased mucus production and decreased free radicals formation in the body. These effects are also associated with the elevated amount of PGE_2_ and SOD activity as well as reduced lipid peroxidation process.

## Figures and Tables

**Table 1:** Effect of *C. xanthorrhiza* leaf extract on the biochemical parameter of renal function test in experimental rats.

Dose (5 mL/kg)	Sodium (mM/L)	Potassium (mM/L)	Chloride (mM/L)	CO_2_ (mM/L)	Anion gap (mM/L)	Urea (mM/L)	Creatinine (µM/L)
Vehicle (CMC)	138.67 ± 0.95	6.13 ± 0.13	97.83 ± 0.83	31.28 ± 0.69	15.67 ± 0.49	6.58 ± 0.32	37.50 ± 1.95
LD (2 g/kg)	138.50 ± 0.76	6.40 ± 0.32	98.83 ± 31.5	31.50 ± 0.54	14.50 ± 0.43	6.05 ± 0.51	38.17 ± 1.83
HD (5 g/kg)	138.17 ± 0.70	5.36 ± 0.09	98.83 ± 0.70	30.57 ± 1.73	14.40 ± 0.60	5.52 ± 0.24	38.17 ± 4.05

**Table 2:** Effect of *C. xanthorrhiza* leaf extract on the biochemical parameter of liver function test in experimental rats.

Dose (Male)	Total protein (g/L)	Albumin (g/L)	Globulin (g/L)	TB (µM/L)	CB (µM/L)	AP (IU/L)	ALT (IU/L)	AST (IU/L)	GGT (IU/L)
Vehicle (CMC)	64.83 ± 1.25	11.83 ± 0.48	60.00 ± 1.65	2.00 ± 0.00	1.00 ± 0.00	415.50 ± 33.88	89.67 ± 7.90	213.00 ± 3.21	3.50 ± 0.50
LD (2 g/kg)	64.17 ± 1.44	11.00 ± 0.37	60.17 ± 1.22	2.40 ± 0.24	1.00 ± 0.00	411.67 ± 69.17	89.17 ± 5.99	212.83 ± 10.16	3.33 ± 0.33
HD (5 g/kg)	64.00 ± 1.93	11.83 ± 0.60	60.17 ± 2.04	2.60 ± 5.60	1.00 ± 0.00	396.00 ± 16.86	88.17 ± 4.75	214.17 ± 4.57	4.50 ± 0.50

The data expressed as mean ± S.E.M (*n* = 6). There are no significant differences between group and control, significant at *P* value < 0.05 using one-way ANOVA. LD: low dose; HD: high dose; TB: total bilirubin; CB: conjugated bilirubin; AP: alkaline phosphatase; ALT: alanine aminotransferase; AST: aspartate aminotransferase; GGT: G-glutamyl transferase; TG: triglycerides; TC: total cholesterol; HDL C: high density lipoprotein cholesterol.

**Table 3:** Effect of *C. xanthorrhiza* leaf extract on formation of ulcer area, percentage of inhibition, mucus weight, and amount of prostaglandin E_2_ (PGE_2_), superoxide dismutase (SOD), and malondialdehyde (MDA) substances in rats.

Group	Pretreatment (5 mL/kg)	Posttreatment	Ulcer area (mm^2^) mean ± SEM	Inhibition (%)	Mucus weight (g)	PGE_2_ (pg/mL)	SOD (U/mL)	MDA (nmol/L)
Normal	CMC	CMC	—	—	0.59 ± 0.031	128.00 ± 0.06	0.552 ± 0.02	17.4 ± 0.008
Ulcer control	CMC	Absolute ethanol	850.00 ± 14.43	—	0.23 ± 0.015	45.50 ± 0.002	0.206 ± 0.03	59.2 ± 0.03
Positive	Omeprazole (20 mg/kg)	Absolute ethanol	178.00 ± 9.60	79.06 ± 0.21	0.46 ± 0.022	97.96 ± 0.006	0.527 ± 0.004	32.58 ± 0.002
Extract	Low dose of extract (250 mg/kg)	Absolute ethanol	0.00 ± 0.00	100.00 ± 0.00	0.37 ± 0.015	81.09 ± 0.04	0.411 ± 0.1	39.7 ± 0.02
Extract	High dose of extract (500 mg/kg)	Absolute ethanol	0.00 ± 0.00	100.00 ± 0.00	0.49 ± 0.012	93.52 ± 0.07	0.461 ± 0.007	25.88 ± 0.01
